# JKK-1(3E), a JKK-1 mutant with predicted phosphomimetic amino acid substitutions

**DOI:** 10.17912/micropub.biology.000785

**Published:** 2023-03-24

**Authors:** Inka Busack, Henrik Bringmann

**Affiliations:** 1 TU Dresden, Dresden, Saxony, Germany

## Abstract

Phosphomimetic substitutions have been instrumental in understanding the role of phosphorylation in protein function. Here we describe the design and construction of a predicted phosphomimetic allele of the JUN kinase kinase gene *jkk-1* in *C. elegans.* To generate the phosphomimetic kinase mutant JKK-1(3E), we edited *jkk-1* to introduce three amino acid substitutions, S274E, S278E and S280E. The resulting strain is homozygous viable and extends the survival of L1-arrested larvae. This survival-extending phenotype suggests that the phosphomimetic mutations might promote activation of JKK-1 during the arrest. This *jkk-1* potential gain-of-function allele might be useful for studying the regulation and functions of JKK-1.

**Figure 1. Design of a predicted phosphomimetic allele of jkk-1:**
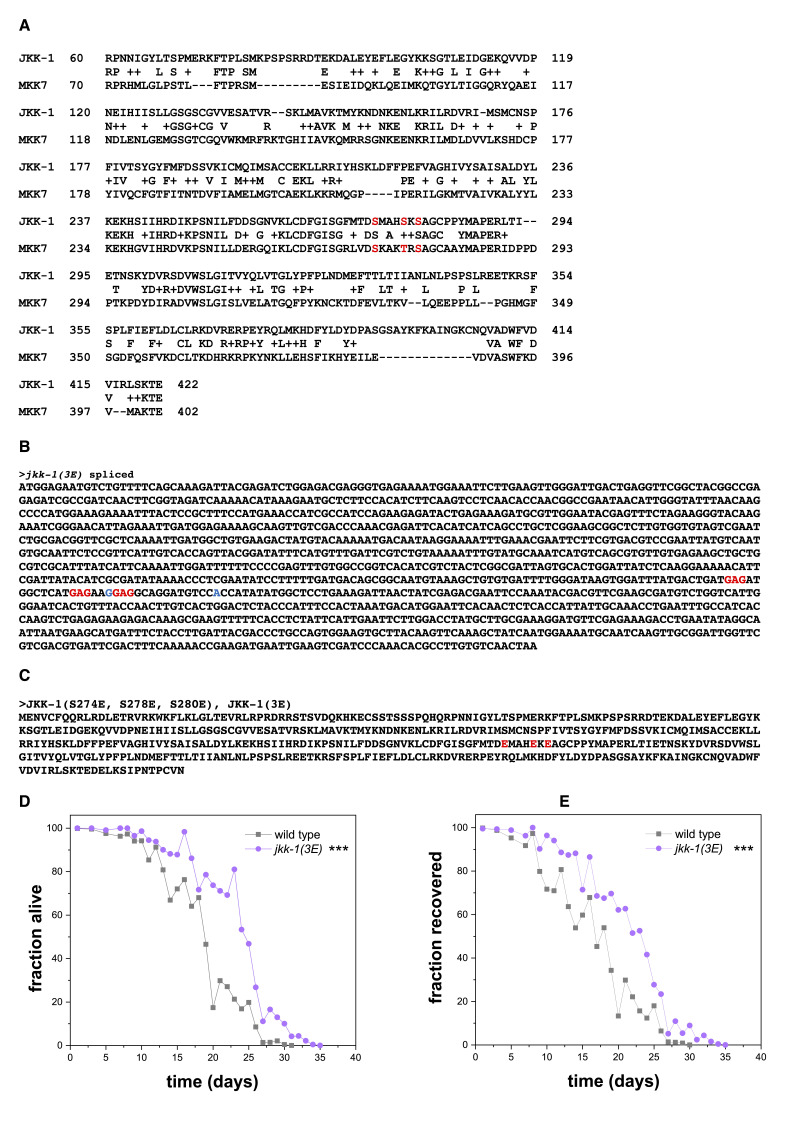
A) Alignment of JKK-1 and MKK7. MKK7 residues S271, T275, and S277 and the homologous JKK-1 residues S274, S278, and S280E are labelled in red. B) Spliced *jkk-1(3E)* gene with the phosphomimetic mutations labelled in red, synonymous changes to prevent recutting of Crispr-Cas9 are indicated in blue. C) JKK-1(3E) protein sequence with the phosphomimetic mutations labelled in red. D) Worms with phosphomimetic *jkk-1(3E)* have significantly increased survival compared to wild-type worms in L1 arrest. Fisher’s Exact Test was conducted at day 19, when 50% of wild-type worms were alive. ***p=0.00011596. Data includes three technical replicates. E) Recovery from L1 arrest of *jkk-1(3E)* worms is increased compared to wild-type worms. Fisher’s Exact Test was conducted when 50% of wild-type worms were alive (day 19)). ***p=7.23*10 ^-20^ . Data includes three technical replicates.

## Description

Many proteins are regulated by phosphorylation at serine, tyrosine and threonine amino acids. Phosphorylation introduces both negative charges as well as steric alterations to the protein, thus changing its function. Interfering with the phosphorylation system of a protein can be instrumental in understanding its function. An example for manipulation of phosphorylation-based protein function is the introduction of phosphomimetic substitutions, by which amino acids that are phosphorylated as part of the physiological control of this protein are substituted for negatively charged amino acids. For example, serine can be replaced by aspartic acid or glutamic acid, thus constitutively introducing a negative charge at this residue. While phosphorylated serine is similar to aspartic acid and glutamic acid, the degree of similarity is limited, as these substitutions do not fully recapitulate the formal charges and the hydration layer caused by serine phosphorylation (Perez-Mejias et al., 2020).

The JNK Kinase JKK-1 in *C. elegans* is part of the JUN kinase phosphorylation signaling pathway that is involved in multiple physiological process including stress responses(Wolf et al., 2008) and locomotion(Villanueva et al., 2001).

Here we designed and created an allele of *jkk-1* that causes three phosphomimetic substitutions by copying a design that had been previously established in mammals. The mammalian homolog of JKK-1 is MKK7. A constitutively active MKK7 mutant has been constructed by replacing S271, T275, and S277 with glutamic acid (MKK73E) (Holtmann et al., 1999). We used Protein BLAST to identify the homologous amino acids in JKK-1. The BLAST alignment suggested that the homologous amino acids in JKK-1 are S274, S278, and S280E (Figure 1A).

We in silico introduced DNA substitutions for all three of these serine codons to replace them with glutamic acid codons. For this editing, GAG was chosen as the codon, as this codon is optimal for translation (Redemann et al., 2011). These substitutions should result in a phosphomimetic version of JKK-1, JKK-1(S274E, S278E, S280E), or JKK-1(3E). These substitutions are labelled in red in Figure 1B-C).

The endogenous locus of *jkk-1* was edited using Crispr-Cas9 by a commercial service provider (Sunybiotech) to introduce these gene edits, resulting in the allele *jkk-1(syb3225)* . Synonymous mutations were also introduced for the process of gene editing, to prevent recutting by the nuclease during the editing procedure (synonymous changes are labelled in blue in Figure 1B). The final sequence of the gene can be seen in Figure 1B. The allele was Sanger sequenced, which confirmed that the edits had been introduced as designed. The strain carrying *jkk-1(syb3225)* (PHX3225) appears superficially normal and is homozygous viable.

JUN kinase signaling and JKK-1 are required for the activation of the FoxO transcription factor DAF-16, and loss-of-function mutation of *jkk-1* reduces adult lifespan (Oh et al., 2005). FoxO activation is also crucial for survival of L1 larvae during starvation-induced developmental arrest (Baugh and Sternberg, 2006; Koutsoumparis et al., 2022). We hence tested survival of JKK-1(3E) worms during L1 arrest by hatching the worms in the absence of food. JKK-1(3E) worms had an increased survival in L1 arrest (Figure 1D) and also had a higher chance to recover from the arrest upon addition of food and to develop to fertile adults compared to wild type (Figure 1E). These results suggest that JKK-1(3E) might confer a gain-of-function phenotype during L1 arrest that is likely caused by increased JKK-1 activity. Additional experiments should be carried out to further characterize the effects of JKK-1(3E) and to more directly test the gain-of-function phenotype during different stages and conditions. The most direct assessment of JKK-1 activity would be to examine the phosphorylation of its target JNK-1, using available phospho-JNK antibodies (Oh *et al.* , 2005). The next most direct method would be to examine DAF-16 phosphorylation and/or nuclear localization, using commonly available reagents.

The strain PHX3225 carrying JKK-1(3E) is available at the CGC and it should be useful for further studying the functions of JKK-1.

## Methods

The survival assay was carried out as described previously (Koutsoumparis *et al.* , 2022; Wu et al., 2018). Briefly, worms were synchronized via hypochlorite treatment and kept in 2ml Eppendorf tubes in 1ml M9 buffer without any food in a rotator at 20°C. L1 arrested worms were tested for survival and recovery every 2 ^nd^ day for the first 9 days and then every day. For this test, approximately 100 larvae were transferred with a pipette onto a NGM plate seeded with OP50 bacteria and it was counted manually how many worms were alive and dead. Three to five days later, the plate was scored again and we counted how many worms had reached the adult stage. The experiment was performed as three technical replicates. Data from all three replicates were combined by adding all worms together. For statistical testing, Fisher’s Exact Test was conducted on day 19, when approximately 50% of wild-type worms were still alive.

## Reagents

N2: wild type

PHX3225: *jkk-1(syb3225)*
